# Association between aspirin use and decline in intrinsic capacity among community-dwelling elderly: a study based on the Lianyungang ICOPE pilot project

**DOI:** 10.3389/fmed.2026.1769131

**Published:** 2026-06-24

**Authors:** Yifang Wang, Lin Ma, Li Wang, Jing Gao, Rongli Lu, Miao Wen, Shixing Song, Quandong Wang, Yan Dong

**Affiliations:** 1 Department of Psychiatry and Department of Geriatrics, The Zigong Affiliated Hospital, Southwest Medical University, Mental Health Center, Zigong Institute of Brain Science, Zigong, Sichuan, China; 2 Department of Medical Innovation and Research, Chinese PLA General Hospital, Western Medical Branch of PLA General Hospital, Chinese PLA Medical School, Beijing, China; 3 Department of Geriatrics, The Affiliated Lianyungang Hospital of Xuzhou Medical University, Lianyungang Clinical College of Nanjing Medical University, The First People’s Hospital of Lianyungang, Lianyungang, Jiangsu, China; 4 Puxi Community Health Service Center, Lianyungang, Jiangsu, China; 5 People’s Hospital of Guanyun, Lianyungang, Jiangsu, China

**Keywords:** angiotensin-converting enzyme inhibitors/angiotensin II receptor blockers (ACEI or ARBs), aspirin, older adults, intrinsic capacity (IC), sensory function

## Abstract

**Background:**

This study examines the connection between the substantial reduction in intrinsic capacity (IC) and unfavorable outcomes across a number of IC subdomains among older adults living in Chinese communities and the use of aspirin, both alone and in combination with angiotensin-converting enzyme inhibitors/angiotensin II receptor blockers (ACEI or ARBs).

**Methods:**

A 1:1 closest-neighbor propensity score matching (PSM) strategy was used to reduce baseline confounding factors across aspirin users and non-users using cross-sectional data from the Lianyungang ICOPE pilot trial. Multivariate logistic regression analysis was used to evaluate the relationship between aspirin use and a significant deterioration in IC as well as unfavorable outcomes in several IC subdomains (sensory, physical, neuropsychiatric, and nutritional function).

**Results:**

A total of 250 participants were analyzed after propensity score matching. The likelihood of a severe loss of intrinsic capacity was not substantially correlated with aspirin use alone (adjusted odds ratio [OR] = 1.54, 95% confidence interval [CI]: 0.88–2.71, *p* = 0.134). However, aspirin use was strongly linked to negative outcomes in sensory function, including very poor vision (adjusted OR = 2.95, 95% CI: 1.10–7.94, *p* = 0.032), poor hearing (adjusted OR = 3.21, 95% CI: 1.29–7.97, *p* = 0.012), and very poor hearing (adjusted OR = 2.08, 95% CI: 1.02–4.24, *p* = 0.045), all of which were statistically significant (*p* < 0.05).

**Conclusion:**

Aspirin use was not significantly correlated with negative outcomes in the categories of nutritional function, neuropsychiatry, or physical health. The incidence of severe loss of intrinsic ability was significantly reduced when aspirin was used in conjunction with angiotensin-converting enzyme inhibitors (ACEI) or angiotensin receptor blockers (ARBs) (*p* < 0.05). Furthermore, there was a sex variability in the relationship between aspirin use and sensory function-related outcomes.

## 1 Introduction

With the rapid progression of global population aging, projected to encompass 2.0 billion individuals over the age of 60 by 2050 (1), health issues among the elderly have emerged as a central challenge in public health (2). The World Health Organization (WHO) has introduced the concept of Intrinsic Capacity (IC) as a comprehensive framework for health assessment, which includes five fundamental dimensions (3): physical capacity, cognitive function, psychological state, sensory function, and metabolic health (4). Recent data from the WHO Integrated Care for Older People (ICOPE) pilot project in China indicate that 41.2% of older adults residing in the community exhibit impaired intrinsic capacity (5), such impairment is strongly associated with increased dependence on daily activities and a higher risk of mortality. Therefore, identifying modifiable risk factors to delay or mitigate the decline in IC is critical for promoting healthy aging and reducing the burden on healthcare systems.

Aspirin, a widely used antiplatelet agent, is well established for the secondary prevention of cardiovascular disease (6–8). Emerging evidence suggests that aspirin may also exert pleiotropic effects, such as anti-inflammatory and antioxidant actions, which could mitigate age-related functional decline (9). However, recent large-scale clinical trials have prompted shifts in international guidelines, which no longer routinely recommend the use of aspirin for primary prevention in older adults (10). This has efforts to identify appropriate clinical contexts for its use and carefully weigh the benefits against the risks in this population. However, the utilization rate of aspirin for secondary prevention among Chinese cardiovascular disease (CVD) patients aged 40–69 is 51.1% (6). Given the widespread use of aspirin in the elderly population in China, it is imperative toconduct research based on local populations to clarify the net benefitrisk Balance in specific elderly Chinese subgroups, thereby providing targeted guidance for clinical practice.

Despite the growing interest in the potential benefits of aspirin, such as its anti-inflammatory and antioxidant properties, evidence regarding its effects on comprehensive health outcomes, particularly intrinsic capacity (IC), remains limited. Moreover, older adults frequently present with multiple chronic conditions that necessitate the concomitant use of other medications, such as angiotensin-converting enzyme inhibitors (ACEI) or angiotensin receptor blockers (ARBs). These agents are commonly prescribed for comorbidities, including hypertension, coronary artery disease, and heart failure. However, whether the combination of aspirin and ACEI or ARBs exerts synergistic or modifying effects on the functional status of older adults is largely unknown. Previous studies have predominantly focused on single-drug exposures or isolated outcomes, such as cardiovascular events or cognitive decline (11, 12), with limited attention to the joint effects of these medications on IC from a holistic health perspective.

To address these gaps, the present study focused on community-dwelling older adults in Lianyungang, China, and investigated the association between aspirin use and overall IC, as well as its constituent domains. We further evaluated whether concomitant use of ACEI or ARBs modified this association. The main contributions of this study are threefold: 1 it provides novel evidence on the relationship between aspirin and multidimensional IC, addressing a critical gap in the literature; 2 it examines potential synergistic risks associated with combined medication use, offering clinically relevant insights for personalized therapeutic decision-making; 3 it leverages local real-world data to inform the application of international guidelines to the older Chinese population. By elucidating the complex interplay between commonly prescribed medications and functional health, this study aims to provide a stronger evidence base for rational drug use and optimized comprehensive health management in older adults, ultimately supporting the goal of healthy aging in this population.

## 2 Materials and methods

### 2.1 Study design and population

#### 2.1.1 Study design

This cross-sectional study was based on data from the World Health Organization’s Integrated Care for Older People (ICOPE) pilot program conducted in Lianyungang City, China. Between March and April 2024, community-dwelling older adults and those residing in long-term care facilities for at least six months were recruited using a multistage sampling strategy in collaboration with local civil affairs departments and subdistrict offices.

#### 2.1.2 Inclusion and exclusion criteria

**Figure 1:**
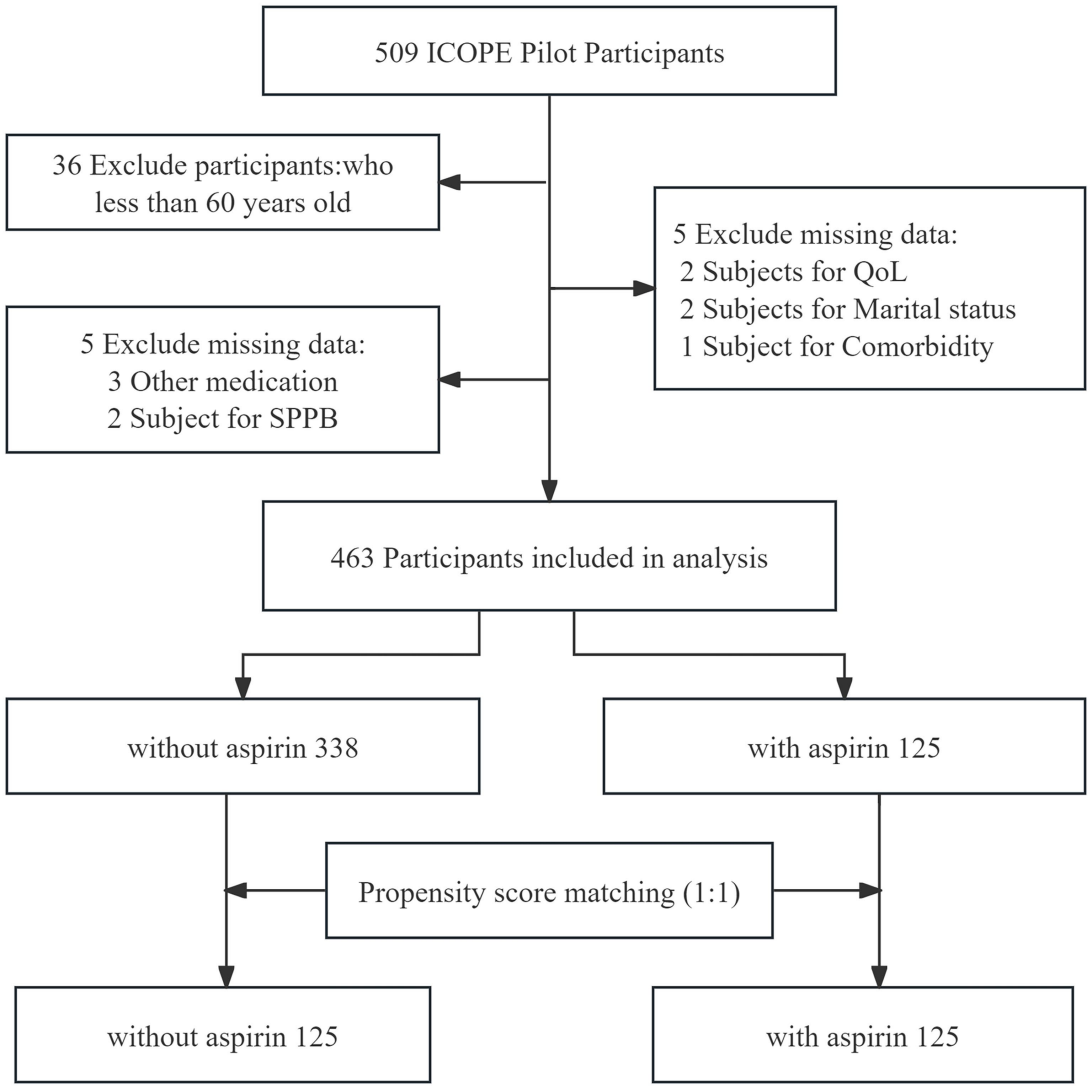
Illustrates the selection process. Of the 509 ICOPE pilot participants, 48 were excluded, leaving 250 for final analysis. QoL, Quality of Life; SPPB, Short Physical Performance Battery.

Eligible participants were aged ≥ 60 years, capable of completing all assessment items independently or with the help of a guardian, and provided written informed consent. Individuals aged < 60 years, those with life-threatening conditions, and participants with missing key variables were excluded. Missing data were addressed using appropriate statistical methods (Figure 1).

#### 2.1.3 Sample size estimation

The target sample size was calculated based on a recommended ratio of 10 to 20 participants per independent variable, with an additional 10% allowance for potential nonresponse. This yielded an estimated requirement of 134–267 participants. Ultimately, 250 eligible individuals were enrolled, exceeding the minimum requirement and ensuring adequate statistical power for planned analyses.

#### 2.1.4 Ethical approval

This study adhered to the Strengthening the Reporting of Observational Studies in Epidemiology (STROBE) guidelines and the principles of the Declaration of Helsinki. Ethical approval was obtained from the Ethics Committee of the First People’s Hospital of Lianyungang (Approval No. QT-20221118001-02; December 9, 2022). All participants provided written informed consent, and all data were anonymized prior to analysis.

#### 2.1.5 Data collection and quality control

Data were collected by trained investigators using a pretested structured questionnaire and validated instruments. Comprehensive quality control measures were implemented throughout the study, including double independent data entry, logical consistency checks, outlier detection, etc.

### 2.2 Construction of the aspirin use variable

Aspirin use was assessed by trained geriatric professionals using a structured questionnaire. Participants were asked to report all medications taken daily for the preceding 3 months. Aspirin use was coded as a binary variable (0 = non-user, 1 = user) (13).

### 2.3 Outcome indicators

IC, defined as the composite of an individual’s physical and mental capacities, is a core indicator of healthy aging (4, 14, 15). IC was evaluated following the World Health Organization’s Integrated Care for Older People (ICOPE) guidelines and handbook (16). It was assessed using the World Health Organization’s ICOPE screening tool, which covers six domains: cognition, locomotion, vitality (nutrition), psychological state (depressive symptoms), vision, and hearing (4, 17, 18). Which recommends a two-step approach. An initial screening was conducted using brief self-reports and objective tests across six domains: cognition, mobility, nutrition, vision, hearing, and psychological status of the patient. When abnormalities were detected, a comprehensive assessment was performed using validated, standardized instruments for each domain. The specific tools employed are described as follows. In line with the tool’s design, a lower total score on this scale indicates a higher level of intrinsic capacity.

#### 2.3.1 Cognitive function

Cognitive decline was initially screened using a two-item test assessing temporal and spatial orientation and a three-word recall task. Participants who failed either component underwent further evaluation with the Mini-Mental State Examination (MMSE) (19). The MMSE has demonstrated good reliability and structural validity in Chinese community-dwelling older adult populations (20), The MMSE provides a total score ranging from 0 to 30, with lower scores indicating greater cognitive impairment. Diagnosis of dementia was based on education-specific cut-off points: a score of ≤17 for individuals with no formal education, ≤20 for those with primary education, ≤22 for those with secondary education (including vocational training), and ≤23 for those with tertiary education (including junior college) (21).

#### 2.3.2 Short physical performance battery (SPPB)

The Short Physical Performance Battery (SPPB) comprises three distinct components: a balance assessment, a 4-meter gait speed test, and a five-repetition sit-to-stand test. The SPPB has demonstrated good reliability and validity in older adult populations (22). Each component is scored on a scale of 0 to 4, resulting in a total score that ranges from 0 to 12. A total score between 0 and 9 indicates impaired mobility (23–25).

#### 2.3.3 Nutritional status

During the assessment of nutritional status, participants will be asked two key questions: Weight Change: “Have you experienced an unintentional weight loss of more than 3 kilograms in the past three months?” Appetite Change: “Have you had a significant decrease in appetite?” A “yes” response to either question will trigger a more comprehensive nutritional screening. Nutritional status was evaluated using the Mini Nutritional Assessment Short-Form (MNA®-SF), which has a total possible score of 14 points (26). Scores ≥ 11 indicate adequate nutritional status, while scores < 11 suggest possible malnutrition. This instrument has been extensively validated in community-dwelling older adult populations, demonstrating acceptable reliability and good diagnostic performance (27).

#### 2.3.4 Vision assessment

For participants with a positive screening result, visual function was further assessed using a standardized module from the comprehensive geriatric assessment, a tool widely applied in major medical institutions in China (28)^.^ This instrument has demonstrated good retest reliability and acceptable concurrent validity. Participants were surveyed regarding visual difficulties during activities (e.g., reading, walking, watching television) and any visual field obstructions or distortions, with responses documented as “yes” or “no.” Using the original scale, scores of ≤1, 2, and 3 represented poor, fair, and good vision, respectively (29). In the revised scale, scores of 1–3, 4, and 5 indicate poor, fair, and good vision, respectively.

#### 2.3.5 Hearing assessment

Hearing function was assessed using a standardized module from the Comprehensive Geriatric Assessment Battery, consistent with the vision assessment tool described above (28), this module has demonstrated good reliability and validity. Hearing ability was assessed using methods including the whisper test, audiometric screening (with a threshold of ≤35 dB), or automated audiometry (30). The assessment comprised three questions: (1) Do others frequently complain that you set the television volume too high? (2) Do you often need others to repeat themselves during conversations (3) Do you have difficulty hearing during phone calls Hearing status was categorized based on the number of affirmative responses: normal Participants who did not pass this initial assessment were further asked about how often they needed others to repeat themselves or had difficulty hearing during telephone conversations. Responses were recorded as “yes” or “no.” According to the original scoring system, a score of ≤1 indicated poor hearing, 2 indicated fair hearing, and 3 indicated good hearing. In the revised scale, however, scores of 1–3 are classified as poor hearing, a score of 4 as fair hearing, and a score of 5 as good hearing.

#### 2.3.6 Psychological status

Psychological capacity was assessed following the ICOPE guidelines, which emphasize screening for depressive symptoms, this module has demonstrated good reliability and validity (16). According to the recommended criteria, participants were classified as having depressive symptoms (coded as 1) if they reported experiencing at least one of the following symptoms“, low mood,” “feeling depressed,” “feeling hopeless,” or “lack of interest or pleasure”, for a minimum of two days during the preceding two weeks. Participants who did not meet these criteria were classified as having a normal psychological status (coded as 0) (31). Affirmative responses to any of these items triggered further assessment with the 5-item Geriatric Depression Scale (GDS-5). A GDS-5 score of ≥2 was defined as indicative of depressive symptoms.

#### 2.3.7 Scoring for cognitive, mobility, nutritional, and depression domains

Each domain was scored using either binary or ternary scoring systems. Most domains were assigned 0 for normal and 1 for abnormal, while vision and hearing used a three-tier scale: 0 for normal, 1 for fair, and 2 for poor. The total intrinsic capacity score was calculated as the sum of all domain scores, ranging from 0 to 8. Based on the total score, intrinsic capacity was categorized as follows: 0–1 indicated high capacity, 2–3 indicated moderate capacity, and ≥4 indicated low capacity.

### 2.4 Covariates

Demographic Information: Sociodemographic covariates included the following: age (categorized as 60–69, 70–79, or ≥80 years); marital status (married and cohabiting, unmarried, widowed, or divorced); educational level (illiterate, primary, secondary [including vocational (training)], or tertiary or higher [including postgraduate education]); and residential status (living in a nursing home or in the community) (Table 1).

**Table 1:** Baseline characteristics before and after propensity score matching.

Before propensity-score matching						After propensity-score matching			
Variables	Total (*n* = 463)	Aspirin (%)		*p* value	SMD 0.1	Total (*n* = 250)	Aspirin (%)		SMD 0.1
		Without (*n* = 338)	With (*n* = 125)				Without (*n* = 125)	With (*n* = 125)	
Age index (years), *n* (%)				0.428	>0.1				<0.1
60–69	6 (1.3)	6 (1.8)	0 (0)						
70–79	180 (38.9)	132 (39.1)	48 (38.4)			96 (38.4)	48 (38.4)	48 (38.4)	
≥80	277 (59.8)	200 (59.2)	77 (61.6)			154 (61.6)	77 (61.6)	77 (61.6)	
Sex, *n* (%)				0.537	<0.1				<0.1
Male	204 (44.1)	146 (43.2)	58 (46.4)			116 (46.4)	58 (46.4)	58 (46.4)	
Female	259 (55.9)	192 (56.8)	67 (53.6)			134 (53.6)	67 (53.6)	67 (53.6)	
Living environment, *n* (%)				0.797	<0.1				<0.1
Community	249 (53.8)	183 (54.1)	66 (52.8)			126 (50.4)	60 (48)	66 (52.8)	
Nursing home	214 (46.2)	155 (45.9)	59 (47.2)			124 (49.6)	65 (52)	59 (47.2)	
Education, *n* (%)				0.125	>0.1				>0.1
Illiterate	156 (33.7)	122 (36.1)	34 (27.2)			80 (32.0)	46 (36.8)	34 (27.2)	
Primary school	141 (30.5)	103 (30.5)	38 (30.4)			74 (29.6)	36 (28.8)	38 (30.4)	
Middle school	80 (17.3)	60 (17.8)	20 (16)			44 (17.6)	24 (19.2)	20 (16)	
High school	53 (11.4)	34 (10.1)	19 (15.2)			32 (12.8)	13 (10.4)	19 (15.2)	
College	18 (3.9)	10 (3)	8 (6.4)			10 (4.0)	2 (1.6)	8 (6.4)	
Master’s degree or above	15 (3.2)	9 (2.7)	6 (4.8)			10 (4.0)	4 (3.2)	6 (4.8)	
Dementia, *n* (%)				0.633	<0.1				<0.1
No	286 (61.8)	211 (62.4)	75 (60)			149 (59.6)	74 (59.2)	75 (60)	
Yes	177 (38.2)	127 (37.6)	50 (40)			101 (40.4)	51 (40.8)	50 (40)	
Malnutrition, *n* (%)				0.358	<0.1				<0.1
No	400 (86.4)	289 (85.5)	111 (88.8)			217 (86.8)	106 (84.8)	111 (88.8)	
Yes	63 (13.6)	49 (14.5)	14 (11.2)			33 (13.2)	19 (15.2)	14 (11.2)	
Vision, *n* (%)				0.022	>0.1				<0.1
Normal	328 (70.8)	250 (74)	78 (62.4)			169 (67.6)	91 (72.8)	78 (62.4)	
Poor	95 (20.5)	65 (19.2)	30 (24)			56 (22.4)	26 (20.8)	30 (24)	
Very Poor	40 (8.6)	23 (6.8)	17 (13.6)			25 (10.0)	8 (6.4)	17 (13.6)	
Hearing, *n* (%)				0.018	>0.1				<0.1
Normal	304 (65.7)	232 (68.6)	72 (57.6)			161 (64.4)	89 (71.2)	72 (57.6)	
Poor	52 (11.2)	30 (8.9)	22 (17.6)			33 (13.2)	11 (8.8)	22 (17.6)	
Very Poor	107 (23.1)	76 (22.5)	31 (24.8)			56 (22.4)	25 (20)	31 (24.8)	
Depression, *n* (%)				0.256	>0.1				>0.1
No	16 (3.5)	14 (4.1)	2 (1.6)			5 (2.0)	3 (2.4)	2 (1.6)	
Yes	447 (96.5)	324 (95.9)	123 (98.4)			245 (98.0)	122 (97.6)	123 (98.4)	
ACEI or ARB, *n* (%)				< 0.001	>0.1				<0.1
No	382 (82.5)	297 (87.9)	85 (68)			192 (76.8)	107 (85.6)	85 (68)	
Yes	81 (17.5)	41 (12.1)	40 (32)			58 (23.2)	18 (14.4)	40 (32)	
Other medications, *n* (%)				< 0.001	>0.1				<0.1
No	280 (60.5)	179 (53)	101 (80.8)			169 (67.6)	68 (54.4)	101 (80.8)	
Yes	183 (39.5)	159 (47)	24 (19.2)			81 (32.4)	57 (45.6)	24 (19.2)	
IC, Median (IQR)	2.0 (1.0, 4.0)	2.0 (1.0, 4.0)	3.0 (2.0, 4.0)	0.016	>0.1	4.0 (2.0, 5.0)	3.0 (2.0, 4.0)	4.0 (3.0, 5.0)	>0.1
Marital status, *n* (%)				0.525	>0.1				>0.1
Single	26 (5.6)	20 (5.9)	6 (4.8)			13 (5.2)	7 (5.6)	6 (4.8)	
Married	217 (46.9)	158 (46.7)	59 (47.2)			118 (47.2)	59 (47.2)	59 (47.2)	
Divorced	8 (1.7)	4 (1.2)	4 (3.2)			5 (2.0)	1 (0.8)	4 (3.2)	
Widowed	212 (45.8)	156 (46.2)	56 (44.8)			114 (45.6)	58 (46.4)	56 (44.8)	
CVD, *n* (%)				0.42	<0.1				<0.1
No	335 (72.4)	248 (73.4)	87 (69.6)			174 (69.6)	87 (69.6)	87 (69.6)	
Yes	128 (27.6)	90 (26.6)	38 (30.4)			76 (30.4)	38 (30.4)	38 (30.4)	
SPPB, *n* (%)				0.072	>0.1				<0.1
No	353 (76.2)	265 (78.4)	88 (70.4)			184 (73.6)	96 (76.8)	88 (70.4)	
Yes	110 (23.8)	73 (21.6)	37 (29.6)			66 (26.4)	29 (23.2)	37 (29.6)	

Data were mean ± SD or median (IQR) for skewed variables or numbers (proportions) for categorical variables. SMD: standardized mean differences. SMD greater than 0.1, indicating cohort imbalance.

*p*-value was based on χ^2^ or analysis of variance or Mann–Whitney U-test where appropriate.

ACEI: Angiotensin-Converting Enzyme Inhibitor. ARB: Angiotensin II Receptor Blocker. CVD: Cardiovascular Disease, which includes CHD: Coronary Heart Disease, heart failure, atrial fibrillation, stroke, and TIA (Transient Ischemic Attack).

### 2.5 Statistical analyses

#### 2.5.1 Descriptive statistics and covariate balance

The baseline characteristics of the participants were summarized separately for the pre- and post-propensity score matching (PSM) cohorts. Continuous variables are presented as mean ± standard deviation (SD) or median (interquartile range, IQR), depending on their distribution, whereas categorical variables are expressed as counts and percentages (n [%]). Prior to matching, between-group differences were assessed using the t-test or Mann–Whitney U test for continuous variables and the chi-square test or Fisher’s exact test for categorical variables. After matching, covariate balance was evaluated using standardized mean differences (SMDs), with an SMDs <0.1 considered indicative of adequate balance (Table 1).

#### 2.5.2 Outcome variable specification and regression modeling

The composite IC score, which ranged from 0 to 8, was initially treated as a continuous variable. However, following PSM, only three participants were classified as having normal IC (score 0–1), precluding a meaningful analysis of the ordinal outcome. Given the clinical relevance of identifying severe IC decline and informed by prior literature and expert consensus, a binary outcome was defined using a cutoff of ≥4 points to indicate low capacity (severe impairment) (28). This threshold was applied to the subsequent regression analyses (Table 2).

**Table 2:** Association between aspirin use and health outcomes after propensity score matching (*n* = 250).

Outcome category	Outcome (reference: reference group)	crude OR (95% CI)	crude *p* value	adj OR (95% CI)	adj P Value
Primary outcome	IC: Moderate or below vs. Severe	1.34 (0.81–2.20)	0.255	1.54 (0.88–2.71)	0.134
Secondary outcome - sensory	Vision: Very Poor vs. Normal	2.48 (1.01–6.05)	0.046∗	2.95 (1.10–7.94)	0.032∗
	Vision: Poor vs. Normal	1.35 (0.73–2.47)	0.337	1.13 (0.58–2.21)	0.725
	Hearing: Very Poor vs. Normal	1.53 (0.83–2.83)	0.171	2.08 (1.02–4.24)	0.045∗
	Hearing: Poor vs. Normal	2.47 (1.12–5.44)	0.024∗	3.21 (1.29–7.97)	0.012∗
Secondary outcome - functional	SPPB: Abnormal vs. Normal	1.39 (0.79–2.45)	0.252	1.51 (0.81–2.81)	0.192
Secondary outcome - comorbidity	Dementia: Yes vs. No	0.97 (0.58–1.6)	0.897	1.32 (0.69–2.51)	0.400
	Malnutrition: Yes vs. No	0.7 (0.34–1.47)	0.352	0.92 (0.40–2.12)	0.843
	Depression: Yes vs. No	0.97 (0.58–1.6)	0.897	1.32 (0.69–2.51)	0.400

IC: Intrinsic Capacity; SPPB: Short Physical Performance Battery; CVD: Coronary Heart Disease, heart failure, atrial fibrillation, stroke, and TIA (Transient Ischemic Attack). OR: Odds Ratio; CI: Confidence Interval. *p*-Value was based on χ^2^ or analysis of variance or Mann–Whitney U-test where appropriate. IC score 0–8; Moderate or below: IC score 0–3; Severe (IC score 4–8). All adjusted ORs were adjusted for age index, gender, education, marital status, living environment, cardiovascular disease, other medications, and ACEI or ARB. ^∗^*p* < 0.05 was considered statistically significant.

To examine the association between asprin use and IC, fully adjusted multivariable logistic regression models were constructed, incorporating all matched covariates to account for residual confounding. In addition to the composite outcome, separate models were fitted for each IC subdomain (cognition, physical mobility, nutrition, vision, hearing, and psychological status). Covariate selection for these models was guided by three complementary criteria: (1) variance inflation factor (VIF) testing to assess multicollinearity, with all VIF values <5.0, indicating no serious multicollinearity; (2) inclusion of clinically relevant variables based on expert consensus; (3) inclusion of potential confounders supported by prior literature. This approach ensured model parsimony while maintaining the validity (Supplementary Table 1).

#### 2.5.3 Subgroup analyses

**Figure 2:**
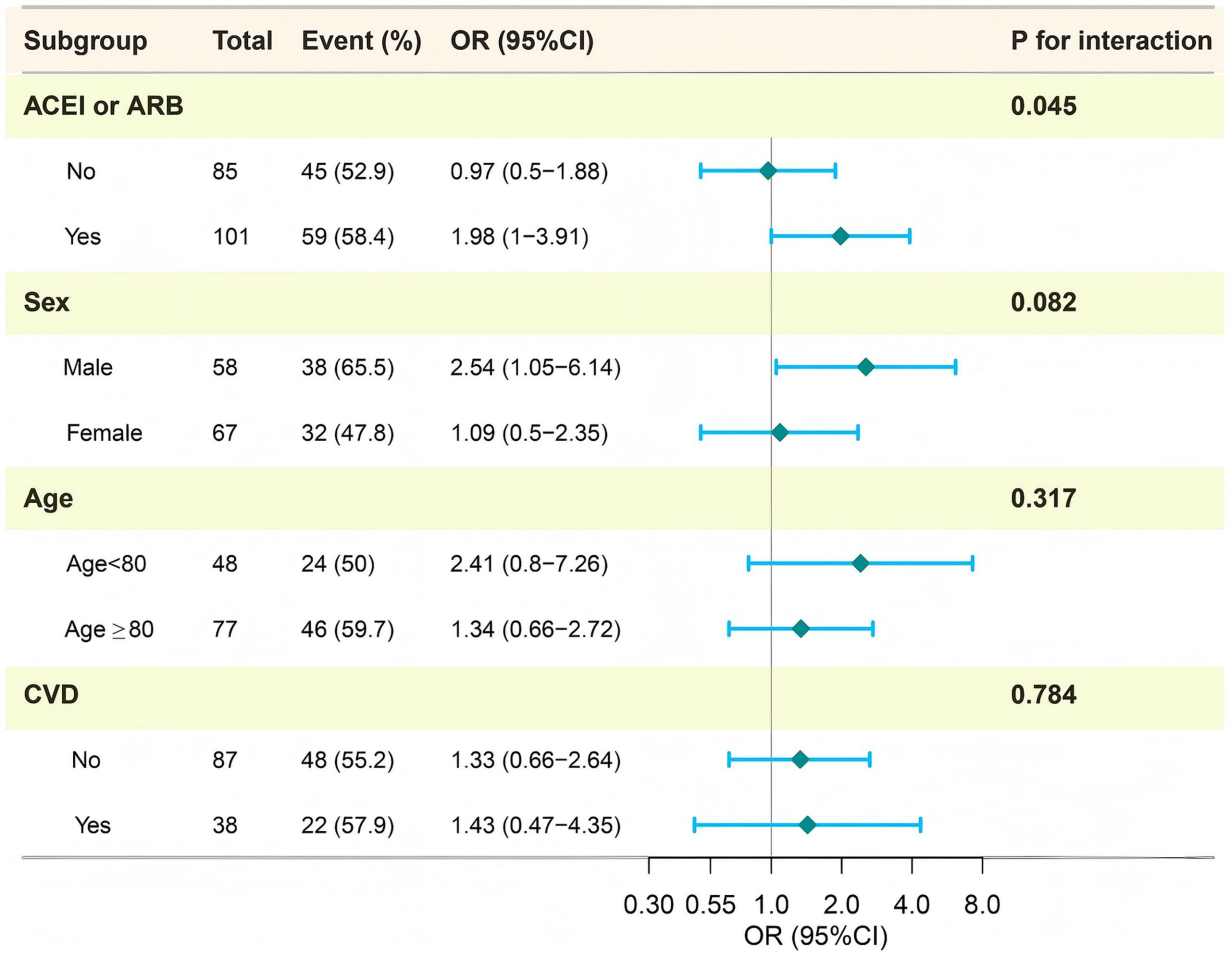
Forest plot. Comparison of the relationship between aspirin as a categorical variable and intrinsic capacity in different subgroups. Stratified analysis to evaluate the effect of aspirin on intrinsic capacity. Results are presented as adjusted odds ratios (95% confidence intervals) of aspirin, which were adjusted for age index, gender, education, marital status, living environment, CVD, other medications.

To evaluate the robustness of the primary findings, subgroup analyses were conducted stratified by age (<80 vs. ≥80 years), sex (male vs. female), cardiovascular disease status (yes vs. no), and ACEI/ARBs use (yes vs. no). Within each stratum, fully adjusted multivariable logistic regression models were fitted to estimate the association between aspirin use and severe IC decline (Figure 2).

#### 2.5.4 Interaction analysis

**Figure 3:**
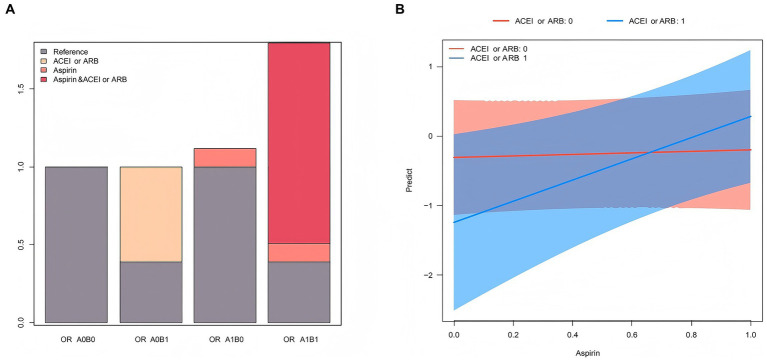
Interaction effect of aspirin and ACEI/ARB use on the risk of IC impairment. **(A)** Bar plot of OR values in four combined groups of aspirin and ACEI/ARB use; **(B)** Line plot of the predictive effect of aspirin use on the outcome in different ACEI/ARB use status. Aspirin (−): 0 = No aspirin use, Aspirin (+) = 1: Aspirin use; ACEI/ARB (−): 0 = No ACEI/ARB use, ACEI/ARB (+) = 1: ACEI/ARBs use; OR = odds ratio. Adjusted for age index, gender, education, marital status, living environment, CVD, other medics.

The joint effects of aspirin and ACEI/ARBs use on IC decline were examined using both additive and multiplicative scales. Additive interaction was assessed using three metrics: relative excess risk due to interaction (RERI), attributable proportion due to interaction (AP), and synergy index (SI). RERI >0 indicates a positive additive interaction (i.e., synergistic effect), with the null hypothesis of RERI = 0. AP represents the proportion of the combined effect attributable to the interaction, with AP = 0 under the null hypothesis. An SI > 1 suggests that the joint effect exceeds the sum of the individual effects, with SI = 1 under the null hypothesis. These measures were calculated to determine whether the risk associated with concurrent aspirin and ACEI/ARBs use exceeded the cumulative risk of each medication used independently (Figure 3; Table 3).

**Table 3:** Interaction effect of aspirin and ACEI/ARB use on the risk of IC impairment.

Subgroup	*N* (%)	OR	95% CI	*p*-value	*P* for interaction
ACEI or ARB (−)					0.045*
Aspirin (−)	107 (55.729)	Reference	—	—	
Aspirin (+)	85 (44.271)	1.086	0.571–2.067	0.802	
ACEI or ARB (+)					
Aspirin (−)	18 (31)	Reference	—	—	
Aspirin (+)	40 (69)	4.657	1.329–16.324	0.016*	
Additive interaction indices					
Multiplicative scale		4.289	1.043–17.63	0.044*	
RERI		1.331	−0.077–2.739	0.032*	
AP		0.738	0.265–1.21	0.001*	

^∗^*p* < 0.05 was considered statistically significant; Aspirin (−): No aspirin use, Aspirin (+): Aspirin use; ACEI or ARB (−): No ACEI/ARB use, ACEI or ARB (+): ACEI or ARB use; OR: odds ratio; CI: confidence interval; RERI: Relative excess risk due to interaction; AP: Attributable proportion; SI=Synergy index; —: no corresponding value. Adjusted for age index, gender, education, marital status, living environment, CVD, other medics.

#### 2.5.5 Sensitivity analysis for unmeasured confounding (E-value)

To assess the potential impact of unmeasured confounding factors on the observed associations, E-values were calculated for the primary outcome of severe IC decline. The E-value quantifies the minimum strength of association that an unmeasured confounder would need to have with both the exposure (aspirin use) and the outcome (severe IC decline), conditional on the measured covariates, to fully explain the observed exposure–outcome association. Higher E-values indicate that stronger unmeasured confounding would be required to negate the findings, thereby providing a measure of the robustness of the results to potential unmeasured confounding (Supplementary Figure 1).

## 3 Results

### 3.1 Baseline characteristics and covariate balance

A total of 463 older adults were included in the propensity score matching (PSM) analysis, of whom 125 (27.0%) were aspirin users and 338 (73.0%) were non-users (Figure 1). In the pre-matching cohort, significant differences were observed between the two groups in terms of IC scores, ACEI/ARBs use, other concomitant medication use, and visual and auditory function (all *p* < 0.05). No significant differences were detected in age, sex, education level, marital status, living environment, or cardiovascular disease prevalence (all *p* > 0.05) (Table 1).

Following 1:1 nearest-neighbor matching with a caliper width of 0.3, a matched cohort of 250 participants (125 per group) was created. With the mean differences (SMDs) for the vast majority of indicators being below 0.1. Notably, variables that differed significantly prior to matching, Covariate balance was substantially improved after matching, including other concomitant medication use, ACEI/ARBs use, and visual and auditory function, demonstrated markedly reduced standardized differences post-matching, confirming that PSM effectively balanced baseline confounders between the two groups (Table 1).

### 3.2 Association between aspirin use and severe IC decline

After propensity score matching to adjust for baseline confounders, a fully adjusted logistic regression model was fitted, including age group, sex, education level, marital status, living environment, cardiovascular disease, other concomitant medication use, and ACEI/ARB use. In this model, aspirin use was not significantly associated with a severe decline in intrinsic capacity (IC score ≥4). Although aspirin users exhibited 54% increased odds of severe IC decline compared with non-users, this finding did not reach statistical significance (adjusted odds ratio [OR] = 1.54, 95% confidence interval [CI]: 0.88–2.71, *p* = 0.134) (Table 2).

In the propensity score-matched cohort, further analyses were conducted to examine the association between aspirin use and adverse outcomes across the key IC subdomains (Table 2). After full adjustment, no significant associations were observed between aspirin use and the risk of dementia (OR = 1.32, 95% CI 0.69–2.51, *p* = 0.400), malnutrition (OR = 0.92, 95% CI 0.40–2.12, *p* = 0.843), or depression (OR 1.32, 95% CI 0.69–2.51, *p* = 0.400).

In the propensity score-matched cohort, after full adjustment for confounders, aspirin use was not significantly associated with overall severe IC decline or impairments in physical function, nutritional status, cognitive function, or psychological status (*p* > 0.05) (Table 2). However, significant associations were observed between aspirin use and specific sensory impairment. Aspirin users exhibited higher odds of moderate visual impairment (OR = 1.13), 95% CI: 0.58–2.21, *p* = 0.725), severe visual impairment (OR = 2.95, 95% CI: 1.10–7.94, *p* = 0.032); moderate hearing impairment (OR = 3.21, 95% CI: 1.29–7.97, *p* = 0.012), and severe hearing impairment (OR = 2.08, 95% CI: 1.02–4.24, (*p* = 0.045) (Table 2). These findings suggest that aspirin use may be differentially associated with sensory function in older adults, requiring further investigation (Table 2).

### 3.3 Subgroup analyses and effect modification

To evaluate the robustness of the association between aspirin use and severe IC decline, subgroup analyses were performed stratified by age (<80 vs. ≥80 years), sex, cardiovascular disease status, and ACEI/ARB use. Fully adjusted logistic regression models were fitted within each stratum, and a forest plot was generated to visualize the effect estimates across subgroups (Figure 2).

A significant interaction was observed between aspirin and ACEI/ARBs use (P for interaction = 0.045). Among participants not using ACEI/ARBs, aspirin use was not significantly associated with severe IC decline (OR = 0.97, 95% CI: 0.50–1.88). In contrast, among ACEI/ARBs users, aspirin use was significantly associated with an increased risk of severe IC decline (OR = 1.98, 95% CI: 1.00–3.91) (Figure 2).

Sex also modified the association between aspirin use and severe IC decline. In men, aspirin use was significantly associated with higher odds of severe IC decline (adjusted OR = 2.54, 95% CI: 1.05–6.14), whereas no significant association was observed in women (adjusted OR = 1.09, 95% CI: 0.50–2.35), suggesting a potential sex-specific effect (Figure 2).

No significant effect modification was detected by age or CVD status (both P for interaction > 0.05). The association between aspirin use and severe IC decline was consistent across age strata (<80 years: OR = 2.41, 95% CI: 0.80–7.26; ≥80 years: OR = 1.34, 95% CI: 0.66–2.72) and CVD status (with CVD: OR = 1.43, 95% CI: 0.47–4.35; without CVD: OR = 1.33, 95% CI: 0.66–2.64), indicating the robustness of the primary findings across these subgroups (Figure 2).

### 3.4 Interaction between aspirin and ACEI/ARBs use on IC decline

To further elucidate the combined effects of aspirin and ACEI/ARBs on severe IC decline, multiplicative and additive interaction analyses were performed (Table 3; Figure 3). Stratified analyses revealed a significant synergistic effect between the two medications. Among participants not using ACEI or ARBs, aspirin use was not significantly associated with severe IC decline (adjusted OR = 1.085, 95% CI: 0.571–2.067, *p* = 0.802). However, among ACEI or ARBs users, concomitant aspirin use was significantly associated with an increased risk of severe IC decline (adjusted OR = 4.657, 95% CI: 1.329–16.324, *p* = 0.016) (Figure 3; Table 3).

The additive interaction measures further supported the synergistic effect. The relative excess risk due to interaction (RERI) was 1.331 (95% CI: −0.077–2.739, (*p* = 0.032)), and the attributable proportion due to interaction (AP) was 0.738 (95% CI: 0.265–1.21, (*p* = 0.001)), indicating that approximately 73.8% of the risk in the dual exposure group could be attributed to the interaction between aspirin and ACEI or ARBs. Multiplicative interaction analysis also demonstrated a statistically significant effect (interaction OR = 4.289, 95% CI: 1.043–17.63, *p* = 0.044). Taken together, these findings suggest that the concurrent use of aspirin and ACEI or ARBs exerts a synergistic effect on the risk of severe IC decline in community-dwelling older adults (Figure 3; Table 3).

### 3.5 Sensitivity analysis for unmeasured confounding (E-value)

To assess the robustness of the primary findings to potential unmeasured confounding factors, E-values were calculated for the association between aspirin use and severe IC decline. In the fully adjusted model, the observed odds ratio for severe IC decline among aspirin users compared to non-users was 1.54 (95% CI: 0.88–2.71), with an E-value of 2.45. This E-value indicates that an unmeasured confounder would need to be associated with both aspirin use and severe IC decline by a risk ratio of at least 2.45 each, conditional on the measured covariates, to fully explain the observed point estimate (Supplementary Figure 1).

## 4 Discussion

This study examined the association between aspirin use and intrinsic capacity (IC) related outcomes in community-dwelling older adults participating in the Lianyungang ICOPE pilot project. The main findings were as follows: (1) aspirin monotherapy was not significantly associated with overall severe IC decline; (2) aspirin use was significantly associated with adverse sensory outcomes, including poor and poor vision, poor hearing, and very poor hearing; but showed no significant associations with physical, neuropsychiatric, or nutritional domains; (3) concurrent use of aspirin and ACEI/ARBs was associated with a synergistic increase in the risk of severe IC decline; (4) The association between aspirin use and severe IC decline exhibits sex-specific differences. To our knowledge, this is the first study to identify the synergistic risk of combined aspirin and ACEI or ARB therapy on IC decline in Chinese community-dwelling older adults, providing novel evidence to inform personalized medication management in this population.

### 4.1 Aspirin monotherapy and overall severe IC decline

Our study did not identify a statistically significant association between aspirin monotherapy and severe IC decline (adjusted OR = 1.54, 95% CI: 0.88–2.71, *p* = 0.134). After propensity score matching, baseline confounders, including cardiovascular disease and concomitant medication use, were well balanced between the groups. The calculated E-value of 2.45, indicating that the findings are reasonably robust to potential unmeasured confounding.

These results are consistent with those of several recent studies. Shah et al. (32), in a 10-year follow-up of older U. S. adults, reported no significant long-term effect of aspirin on a composite outcome of death, dementia, or persistent physical disability (HR = 1.01, 95% CI: 0.95–1.08). Handono et al. (33) found that aspirin neither ameliorated nor exacerbated frailty severity in older Australian adults. A systematic review by Ewbank et al. (34) also observed no significant association between aspirin use and adverse outcomes, such as subarachnoid hemorrhage.

In contrast, a subsequent analysis of the ASPREE trial by Handono et al. (33) demonstrated that low-dose aspirin significantly increased the risk of major bleeding events, including intracranial hemorrhage, in healthy older adults aged ≥70 years (HR = 1.38, 95% CI: 1.18–1.62). Several factors may explain the discrepancy between these findings and our neutral result. First, the study endpoints differed fundamentally: the ASPREE trial focused on acute adverse clinical events directly related to aspirin’s antiplatelet effects (e.g., major bleeding, cardiovascular events, and mortality) (34), whereas our study examined IC, a chronic, multifactorial outcome less likely to be directly influenced by a single medication. Second, the baseline health status and inclusion criteria varied considerably. The ASPREE trial enrolled healthy older adults without cardiovascular disease, a population with no clear indication for aspirin use and a potentially greater susceptibility to adverse effects. In contrast, our study included 30.4% of participants with comorbid CVD and 23.2% using ACEI/ARB, suggesting that some participants had clinical indications for the use of aspirin. In such cases, the therapeutic benefits may partially offset the adverse effects, potentially explaining the null findings for the overall impairment of IC decline. Third, differences in the follow-up duration and observation windows may have contributed to the divergent results. The ASPREE trial’s shorter follow-up period is better suited to capture acute bleeding events, whereas impairment of IC develops gradually. Although our study employed PSM to control for baseline confounding, its cross-sectional design precluded the assessment of the long-term cumulative effects of aspirin, which may also explain the lack of a significant association.

### 4.2 Association with severe visual impairment

The significant association between aspirin use and severe visual impairment observed in our study is consistent with a systematic review and meta-analysis by Yan et al. (35), which synthesized data from 16 studies and demonstrated that prolonged aspirin use (>10 years) was significantly associated with an increased risk of age-related macular degeneration (AMD), the leading cause of severe visual impairments in older adults (pooled OR = 2.32, 95% CI: 2.23–2.42, (*p* < 0.01). The results of this investigation indicate that aspirinmediated suppression of the cyclooxygenase COX) pathway may impair connexin-43 (Cx43), mediated gap-junctional intercellular communication (GJIC) in retinal pigment epithelial (RPE) cells. Such disruption compromises the coordinated cellular responses required for the maintenance of outer blood-retinal barrier integrity, ionic homeostasis, and adaptive stress signaling. Consequently, the resultant breakdown in RPE functional synergy precipitates retinal microenvironmental imbalance and promotes photoreceptor degeneration (36). Corroborating this mechanistic insight, Préterre et al. (2025) reported that 76% of patients presenting with acute central retinal artery occlusion, who were administered aspirin as monotherapy, exhibited persistent severe visual impairment (best-corrected visual acuity < 20/400) at one-month follow-up. This clinical observation further underscores the limited capacity of aspirin to preserve neuroretinal function under ischemic conditions, a finding consistent with the proposed compromise in GJIC-dependent RPE cellular coordination (37).

In contrast, Rim et al. (38) used a nationwide Korean cohort of 482,613 individuals and found no significant association between long-term low-dose aspirin use (≤100 mg/day for ≥5 years) and neovascular AMD incidence (adjusted HR = 0.95, 95% CI: 0.71–1.28). Several factors may explain this discrepancy: (1) dosage and duration effects, the association between aspirin and AMD may only manifest with higher doses or longer exposure (>10 years) (39); (2) residual confounding, our study may not have fully adjusted for visual impairment risk factors such as smoking and hypertension; and (3) outcome heterogeneity, neovascular AMD represents only one subtype of vision loss, whereas our “very poor vision” outcome may encompass other aspirin-related pathologies (e.g., retinal vascular occlusion).

#### 4.2.1 Association with hearing impairment

Aspirin-associated hearing impairment exhibits significant dose-dependent and clinical context-dependent characteristics. The underlying mechanisms involve direct cochlear cellular dysfunction and an imbalance in vascular-inflammatory homeostasis within the context of specific systemic diseases (e.g., diabetes).

At the cellular level, high-dose or long-term aspirin use can induce direct ototoxicity by selectively inhibiting prestin, a key motor protein in cochlear outer hair cells, an effect independent of its antiplatelet activity (40).

At the patient level, the risk is modulated by underlying comorbidities. For instance, in individuals with type 2 diabetes and pre-existing inner ear microangiopathy, even low-dose aspirin may increase the risk of sudden sensorineural hearing loss through mechanisms involving insufficient vascular and anti-inflammatory effects (41). This contrasts with findings from studies of healthy older adults, in which low-dose aspirin showed no significant impact on hearing thresholds (42). Highlighting the critical role of background disease in modulating aspirin-related ototoxic risk.

### 4.3 Synergistic effect of aspirin and ACEI/ARB combination

A notable finding of this study was the synergistic effect of concomitant aspirin and ACEI/ARB use on severe hearing decline. This effect builds upon the ototoxicity of aspirin monotherapy and can be understood through an integrated mechanistic framework involving inner ear microcirculatory decompensation and amplification of cellular stress cascades.

First, Synergistic Disruption of Cochlear Microcirculatory Homeostasis: The antiplatelet effect of aspirin and the vasodilatory action of ACEI/ARBs exert compounding effects within the cochlea, particularly in the presence of pre-existing microvascular damage (e.g., due to hypertension or diabetes). Their combination likely disrupts the precise regulation of cochlear capillary perfusion pressure, leading to chronic hypoperfusion and exacerbating hypoxia and nutrient deficiency in outer hair cells and spiral ganglion neurons (43, 44).

Second, exacerbation of Inner Ear Inflammation–Oxidative Stress Axis Imbalance: This extends the “dual insufficiency” hypothesis. Low-dose aspirin provides limited anti-inflammatory action and reduces the synthesis of cytoprotective prostaglandins, while the local anti-inflammatory and antioxidant effects of ACEI/ARBs are also insufficient. Together, the combination may synergistically weaken the endogenous protective capacity of the inner ear, resulting in increased accumulation of reactive oxygen species and release of pro-inflammatory cytokines, thereby accelerating auditory cell damage (40, 45).

Finally, counteraction of Potential Endothelial Protection: Improvements in vascular endothelial function and the potential increase in cochlear protective prostaglandins (e.g., PGI₂) afforded by ACEI/ARBs may be negated by aspirin’s inhibition of cyclooxygenase, thereby theoretically offsetting some of the inner ear protective benefits associated with ACEI/ARB monotherapy (46).

In summary, the combination therapy synergistically targets the vulnerable inner ear microenvironment through multiple pathways, amplifying the risk of hearing impairment, particularly in elderly individuals with pre-existing microvascular disease. This conclusion warrants further validation in large-scale, prospective cohort studies.

### 4.4 Innovations and clinical implications

To our knowledge, this study represents the first investigation to systematically evaluate the association between aspirin use and multidimensional functional outcomes among older adults under the framework of the WHO intrinsic capacity (IC). Our findings indicate a sensory-specific effect of aspirin, with significant associations solely restricted to the visual and auditory domains, whereas no notable impact was observed on physical, neuropsychiatric or nutritional function. This pattern may reflect the unique susceptibility of sensory organs to the pharmacological effects of aspirin, particularly the dense microvasculature and fragile nerve endings within the retina and cochlea. In contrast to single-outcome analyses that merely focus on isolated disorders such as age-related macular degeneration (AMD) or hearing loss, the present study provides a more comprehensive assessment of aspirin safety in the elderly population.

These findings carry substantial clinical implications. Firstly, long-term aspirin users, especially those with comorbid diabetes or vascular diseases, require intensified monitoring of sensory function, including regular vision and hearing screenings, to facilitate early detection of functional impairment. Secondly, low-dose aspirin cannot be deemed universally safe for all older adults, and prescription decisions necessitate a careful balance between cardiovascular benefits and potential sensory system risks. For patients with pre-existing risk factors for visual or auditory impairment (e.g., family history of AMD or inner ear diseases), dose adjustment or substitution with alternative antiplatelet agents may be considered. Individualized risk–benefit stratification coupled with regular functional monitoring remains a core principle when prescribing aspirin to older populations.

### 4.5 Limitations and future directions

Several limitations of this study should be acknowledged. First, information on specific aspirin dosage, duration of use, and frequency of administration was not collected, precluding detailed analysis of dose–response relationships or duration thresholds. Second, although we performed comprehensive adjustments for measured confounders using propensity score matching (PSM) and multivariate regression, and further calculated e-values to evaluate the robustness of our findings, residual confounding due to unmeasured factors, including smoking, alcohol consumption and other lifestyle variables, cannot be completely ruled out. The results demonstrated that only an unmeasured confounder with an extremely strong effect could fully attenuate the observed association between aspirin use and sensory impairment, supporting the relative reliability of our core findings. Third, the cross-sectional design precludes causal inference regarding the relationship between aspirin use and IC decline or impairment. Fourth, the majority of our study population was over 80 years of age, which may limit the generalizability of the findings to other populations.

Future studies should address these limitations through several approaches. First, prospective cohort studies with detailed dose stratification of aspirin are warranted to elucidate the optimal dose and duration thresholds for its effects on sensory function. Second, incorporation of advanced imaging techniques (e.g., optical coherence tomography, electrocochleography) and biomarkers (e.g., serum inflammatory factors, retinal vascular endothelial function indicators) would enable more comprehensive investigation of underlying mechanisms. Third, subgroup analyses in populations with different underlying conditions (e.g., diabetes, cardiovascular disease) would generate more precise evidence to guide personalized medication strategies. Finally, randomized controlled trials examining the impact of aspirin dose adjustment on sensory function and IC in older adults are needed to provide higher-level evidence for clinical guideline development.

## 5 Conclusion

In community-dwelling older adults, aspirin monotherapy was not significantly associated with severe decline in overall intrinsic capacity. However, aspirin use was specifically linked to adverse sensory outcomes, including poor and very poor vision and hearing, with no significant associations observed in physical, neuropsychiatric, or nutritional domains. Concurrent use of aspirin and ACEI/ARBs demonstrated a synergistic effect on the risk of severe IC decline, and the relationship between aspirin and adverse sensory outcomes exhibited sex-specific heterogeneity.

These findings provide empirical evidence to inform individualized aspirin use in older populations. Clinically, decisions regarding aspirin prescription should carefully balance potential cardiovascular benefits against possible sensory function risks, with enhanced monitoring of vision and hearing where indicated. For older adults requiring concomitant ACEI/ARBs therapy, rigorous evaluation of medication necessity and close surveillance of overall intrinsic capacity status are warranted.

## Data Availability

The raw data supporting the conclusions of this article will be made available by the authors, without undue reservation.

## Glossary

IC: Intrinsic Capacity
WHO: World Health Organization
ICOPE: Integrated care for older people
CVD: Cardiovascular disease
MMSE: Mini-Mental State Examination
SPPB: Short Physical Performance Battery
MNA-SF: Mini Nutritional Assessment-Short Form
VIF: Variance Inflation Factor
OR: Odds Ratio
CI: Confidence Interval
ACEI: Angiotensin-Converting Enzyme Inhibitor
ARBs: Angiotensin II Receptor Blocker
CVD: Cardiovascular Disease
PSM: Propensity Score Matching
RERI: Relative Excess Risk due to Interaction
AP: Attributable Proportion
SI: Synergy Index
E-value: Expectation Value
SMD: Standardized Mean Difference
AMD: Age-Related Macular Degeneration
CRAO: Central Retinal Artery Occlusion
SSNHL: Sudden Sensorineural Hearing Loss

## References

[1] Buyl R Beogo I Fobelets M Deletroz C Van Landuyt P Dequanter S . E-health interventions for healthy aging: a systematic review. Syst Rev. (2020) 9:128. doi: 10.1186/s13643-020-01385-8, 32493515 PMC7271471

[2] Yan Y Du Y Li X Ping W Chang Y. Physical function, ADL, and depressive symptoms in Chinese elderly: evidence from the CHARLS. Front Public Health. (2023) 11:1017689. doi: 10.3389/fpubh.2023.1017689, 36923048 PMC10010774

[3] Ma L Chhetri JK Zhang Y Liu P Chen Y Li Y . Integrated Care for Older People Screening Tool for measuring intrinsic capacity: preliminary findings from ICOPE pilot in China. Front Med (Lausanne). (2020) 7:576079. doi: 10.3389/fmed.2020.576079, 33330532 PMC7734133

[4] Koivunen K Schaap LA Hoogendijk EO Schoonmade LJ Huisman M van Schoor NM. Exploring the conceptual framework and measurement model of intrinsic capacity defined by the World Health Organization: a scoping review. Ageing Res Rev. (2022) 80:101685. doi: 10.1016/j.arr.2022.101685, 35830956

[5] Huang ZT Lai ETC Luo Y Woo J. Social determinants of intrinsic capacity: a systematic review of observational studies. Ageing Res Rev. (2024) 95:102239. doi: 10.1016/j.arr.2024.102239, 38382677

[6] Yoo SGK Chung GS Bahendeka SK Sibai AM Damasceno A Farzadfar F . Aspirin for secondary prevention of cardiovascular disease in 51 low-, middle-, and high-income countries. JAMA. (2023) 330:715–24. doi: 10.1001/jama.2023.12905, 37606674 PMC10445202

[7] Jacobsen AP Raber I McCarthy CP Blumenthal RS Bhatt DL Cusack RW . Lifelong aspirin for all in the secondary prevention of chronic coronary syndrome: still sacrosanct or is reappraisal warranted? Circulation. (2020) 142:1579–90. doi: 10.1161/CIRCULATIONAHA.120.045695, 32886529

[8] Grimaldi S Migliorini P Puxeddu I Rossini R De Caterina R. Aspirin hypersensitivity: a practical guide for cardiologists. Eur Heart J. (2024) 45:1716–26. doi: 10.1093/eurheartj/ehae128, 38666370

[9] You M Wang K Pan Y Tao L Ma Q Zhang G . Combined royal jelly 10-hydroxydecanoic acid and aspirin has a synergistic effect against memory deficit and neuroinflammation. Food Funct. (2022) 13:2336–53. doi: 10.1039/d1fo02397g, 35142767

[10] Davidson KW Barry MJ Mangione CM Cabana M Chelmow D Coker TR . Aspirin use to prevent cardiovascular disease: US preventive services task force recommendation statement. JAMA. (2022) 327:1577–84. doi: 10.1001/jama.2022.4983, 35471505

[11] Kingsley J Torimoto K Hashimoto T Eguchi S. Angiotensin II inhibition: a potential treatment to slow the progression of sarcopenia. Clin Sci (Lond). (2021) 135:2503–20. doi: 10.1042/CS20210719, 34751393

[12] Belachew EA Peterson GM Salahudeen MS Radford J Bezabhe WM. Long-term risk of dementia with angiotensin receptor blockers versus angiotensin-converting enzyme inhibitors in hypertensive patients: a 15-year follow-up using the 45 and up study. Geroscience. (2026). doi: 10.1007/s11357-026-02173-3 41760976

[13] Hariri E Matta M Layoun H Badwan O Braghieri L Owens AP . Antiplatelet therapy, abdominal aortic aneurysm progression, and clinical outcomes. JAMA Netw Open. (2023) 6:e2347296. doi: 10.1001/jamanetworkopen.2023.47296 38085542 PMC10716735

[14] Hoogendijk EO Dent E Koivunen K. Intrinsic capacity: an under-researched concept in geriatrics. Age Ageing. (2023) 52:afad183. doi: 10.1093/ageing/afad183, 37782890

[15] Zhou Y Ma L. Intrinsic capacity in older adults: recent advances. Aging Dis. (2022) 13:353–9. doi: 10.14336/AD.2021.0818, 35371613 PMC8947834

[16] Organization WH. Integrated care for older People (ICOPE): Guidance for person - Centred Assessment and Pathways in Primary care. Geneva: World Health Organization (2019).

[17] Maheshwari S Dai C Giri S Harmon C Tucker A Fowler ME . Intrinsic capacity and survival among older adults with gastrointestinal malignancies: the Cancer and aging resilience evaluation registry. Cancer. (2024) 130:3530–9. doi: 10.1002/cncr.35427, 38865419

[18] Gonzalez-Bautista E Llibre-Guerra JJ Sosa AL Acosta I Andrieu S Acosta D . Exploring the natural history of intrinsic capacity impairments: longitudinal patterns in the 10/66 study. Age Ageing. (2023) 52:afad137. doi: 10.1093/ageing/afad137, 37517058 PMC10387229

[19] Verhaar BJH Hendriksen HMA de Leeuw FA Doorduijn AS van Leeuwenstijn M Teunissen CE . Gut microbiota composition is related to AD pathology. Front Immunol. (2021) 12:794519. doi: 10.3389/fimmu.2021.794519 35173707 PMC8843078

[20] Marioni RE Chatfield M Brayne C Matthews FE. The reliability of assigning individuals to cognitive states using the Mini mental-state examination: a population-based prospective cohort study. BMC Med Res Methodol. (2011) 11:127. doi: 10.1186/1471-2288-11-127, 21896187 PMC3175206

[21] van Vliet NA van Heemst D Almeida OP Åsvold BO Aubert CE Bae JB . Association of Thyroid Dysfunction with Cognitive Function: an individual participant data analysis. JAMA Intern Med. (2021) 181:1440–50. doi: 10.1001/jamainternmed.2021.5078, 34491268 PMC8424529

[22] Bidaurrazaga-Letona I Diz JC Torres-Unda J Esain I Monasterio X Zulueta B . Short physical performance battery reliability and validity in adults with mild to moderate intellectual disability. J Intellect Develop Disabil. (2023) 48:238–46. doi: 10.3109/13668250.2023.2166198, 39815921

[23] Martinez-Amezcua P Powell D Kuo PL Reed NS Sullivan KJ Palta P . Association of age-Related Hearing Impairment with Physical Functioning among Community-Dwelling Older Adults in the US. JAMA Netw Open. (2021) 4:e2113742. doi: 10.1001/jamanetworkopen.2021.13742, 34170305 PMC8233700

[24] Exter SH Koenders N Wees P Berg MGA. A systematic review of the psychometric properties of physical performance tests for sarcopenia in community-dwelling older adults. Age Ageing. (2024) 53:afae113. doi: 10.1093/ageing/afae113, 38851214 PMC11162262

[25] Kim JY Gil TH Lee HG Shin JW Jang DH Kim HS . Plasma extracellular vesicles biomarkers linked to lower muscle mass, function and physical performance in sarcopenia. J Cachexia Sarcopenia Muscle. (2025) 16:e13784. doi: 10.1002/jcsm.13784, 40162588 PMC11955922

[26] Rakıcıoğlu N Ulusoy-Gezer HG Çelik B. Compatibility of the Mini nutritional assessment and the healthy diet Indicator in the evaluation of nutritional status in older adults: a community-based study. J Am Coll Nutr. (2025) 44:609–15. doi: 10.1080/27697061.2025.2475879, 40106508

[27] Trampisch US Pourhassan M Daubert D Volkert D Wirth R. Interrater reliability of routine screening for risk of malnutrition with the Mini nutritional assessment short-form in hospital. Eur J Clin Nutr. (2022) 76:1111–6. doi: 10.1038/s41430-022-01080-y, 35194196 PMC9352578

[28] Yun X Wang J Guo AX Gu ZJ Sun Q Xia JM . The association of polypharmacy with intrinsic capacity: an analysis of the WHO ICOPE pilot data from Lianyungang, China. Front Med (Lausanne). (2025) 12:1673885. doi: 10.3389/fmed.2025.1673885, 41426601 PMC12715426

[29] Xu L Li H Li F Zhang T Yan J Yan H . Investigating the trajectories of poor vision in children and adolescents in Wuhan, China from 2016 to 2019: prospective cohort study. JMIR Public Health Surveill. (2025) 11:e53028. doi: 10.2196/53028, 39964957 PMC11855164

[30] Lisan Q Goldberg M Lahlou G Ozguler A Lemonnier S Jouven X . Prevalence of hearing loss and hearing aid use among adults in France in the CONSTANCES study. JAMA Netw Open. (2022) 5:e2217633. doi: 10.1001/jamanetworkopen.2022.17633, 35713903 PMC9206187

[31] Levis B Sun Y He C Wu Y Krishnan A Bhandari PM . Accuracy of the PHQ-2 alone and in combination with the PHQ-9 for screening to detect major depression: systematic review and Meta-analysis. JAMA. (2020) 323:2290–300. doi: 10.1001/jama.2020.6504, 32515813 PMC7284301

[32] Shah RC Ryan J Webb KL Wolfe R Chan A Chong TT . Aspirin and healthy lifespan in older people: main outcome of the ASPREE-XT observational study. Lancet Healthy Longev. (2025) 6:100764. doi: 10.1016/j.lanhl.2025.100764, 41043446 PMC12551462

[33] Handono AP Ryan J Setiawan F Espinoza SE Phyo AZZ Zhu C . The effect of low-dose aspirin on reversing frailty status in older prefrail and frail adults: a secondary analysis of the ASPREE randomised clinical trial. Age Ageing. (2025) 54:afaf271. doi: 10.1093/ageing/afaf271, 40996142 PMC12461696

[34] Ewbank F Birks J Bulters D. A meta-analysis of aspirin and subarachnoid hemorrhage in patients with intracranial aneurysms yields different results to the general population. Int J Stroke. (2022) 17:341–53. doi: 10.1177/17474930211004888 33705214

[35] Yan R Zhao J Zhang X Wang W Jiang Z. Association between aspirin usage and age-related macular degeneration: an updated systematic review and Meta-analysis. Front Pharmacol. (2022) 13:824745. doi: 10.3389/fphar.2022.824745, 35401184 PMC8990128

[36] You L Lin Y Zheng Y Han Z Zeng L Chen H. The impact of aging on ocular diseases: unveiling complex interactions. Aging Dis. (2024) 16:2803–30. doi: 10.14336/AD.2024.0850 39500360 PMC12339180

[37] Wang Y Li Y Feng J Wang C Wan Y Lv B . Transcriptional responses in a mouse model of silicone wire embolization induced acute retinal artery ischemia and reperfusion. eLife. (2024) 13:RP98949. doi: 10.7554/eLife.98949 39382568 PMC11464005

[38] Rim TH Yoo TK Kwak J Lee JS Kim SH Kim DW . Long-term regular use of low-dose aspirin and Neovascular age-related macular degeneration: National Sample Cohort 2010-2015. Ophthalmology. (2019) 126:274–82. doi: 10.1016/j.ophtha.2018.09.014, 30240791

[39] Robman LD Wolfe R Woods RL Thao LTP Makeyeva GA Hodgson LAB . Effect of low-dose aspirin on the course of age-related macular degeneration: a secondary analysis of the ASPREE randomized clinical trial. JAMA Ophthalmol. (2024) 142:627–35. doi: 10.1001/jamaophthalmol.2024.1584, 38780931 PMC11117148

[40] Tzelnick S Mizrachi A Barkan N Shivatzki S Yosefof E Hikri E . The protective effect of aspirin-induced temporary threshold shift in an animal model of cisplatin-related ototoxicity. J Cancer Res Clin Oncol. (2023) 149:2009–16. doi: 10.1007/s00432-022-04144-5, 35773430 PMC11798173

[41] Liu CC Chen WM Shia BC Wu SY Chou WJ. Dose-response relationship of aspirin and sudden sensorineural hearing loss risk in type 2 diabetes: aspirin dosage on SSNHL risk in T2D. Hear Res. (2025) 459:109217. doi: 10.1016/j.heares.2025.109217, 39933255

[42] Clark DPQ Zhou Z Hussain SM Tran C Britt C Storey E . Low-dose aspirin and progression of age-related hearing loss: a secondary analysis of the ASPREE randomized clinical trial. JAMA Netw Open. (2024) 7:e2424373. doi: 10.1001/jamanetworkopen.2024.24373, 39052288 PMC11273233

[43] Alenazy FO Harbi MH Kavanagh DP Price J Brady P Hargreaves O . Amplified inhibition of atherosclerotic plaque-induced platelet activation by glenzocimab with dual antiplatelet therapy. J Thromb Haemost. (2023) 21:3236–51. doi: 10.1016/j.jtha.2023.07.018, 37541591

[44] Natale P Palmer SC Navaneethan SD Craig JC Strippoli GF. Angiotensin-converting-enzyme inhibitors and angiotensin receptor blockers for preventing the progression of diabetic kidney disease. Cochrane Database Syst Rev. (2024) 4:Cd006257. doi: 10.1002/14651858.CD006257.pub2, 38682786 PMC11057222

[45] Zhu XH Jialin Lin X. Experimental observation of sodium salicylate enhancing oxidative stress-mediated damage to cochlear spiral ganglion neurons. Chinese J New Clinical Med. (2023) 16, 447–451.

[46] Becerra Calderon A Shroff UN Deepak S Izuhara A Trogen G McDonough AA . Angiotensin II directly increases endothelial calcium and nitric oxide in kidney and brain microvessels in vivo with reduced efficacy in hypertension. J Am Heart Assoc. (2024) 13:e033998. doi: 10.1161/JAHA.123.033998, 38726925 PMC11179802

