# Supplementary material for: Association between aspirin use and decline in intrinsic capacity among community-dwelling elderly: a study based on the Lianyungang ICOPE pilot project
Source: Front Med (Lausanne). 2026 Jun 24;13:1769131. doi: 10.3389/fmed.2026.1769131 (PMC13341446; doi:10.3389/fmed.2026.1769131)
Supplement: Supplementary file 1 [file Data_Sheet_1.docx]

Supplementary Table 1. Selection and distribution of covariates for multivariate adjustment.

| Term 1 | Coeff 1 | Change percentage | GVIF | DF | GVIF^(1/(2*Df)) |
| --- | --- | --- | --- | --- | --- |
| Crude | 0.41 | Ref. | 2.05 | 1.00 | 1.43 |
| SPPB | 0.29 | -30.10 | 1.15 | 1.00 | 1.07 |
| CVD | 0.40 | -4.50 | 1.21 | 1.00 | 1.10 |
| Gender | 0.41 | -1.00 | 1.26 | 1.00 | 1.12 |
| Nursing home | 0.40 | -2.70 | 1.81 | 1.00 | 1.35 |
| Education | 0.45 | 9.30 | 1.90 | 5.00 | 1.07 |
| Malnutrition | 0.44 | 6.90 | 1.24 | 1.00 | 1.12 |
| Vision | 0.15 | -62.90 | 1.40 | 2.00 | 1.09 |
| Hearing | 0.20 | -51.80 | 1.56 | 2.00 | 1.12 |
| Depression | 0.37 | -11.50 | 1.11 | 1.00 | 1.06 |
| Age index | 0.39 | -5.80 | 1.37 | 2.00 | 1.08 |
| Marital status | 0.43 | 3.50 | 1.87 | 3.00 | 1.11 |
| ACEI or ARB | 0.45 | 9.90 | 1.61 | 1.00 | 1.27 |
| Other medics | 0.47 | 14.40 | 1.82 | 1.00 | 1.35 |

Footnotes:

Abbreviations: SPPB, Short Physical Performance Battery; CVD, Cardiovascular Disease; ACEI,

Angiotensin-Converting Enzyme Inhibitor; ARB, Angiotensin Receptor Blocker; IC, Intrinsic

Capacity.

Crude: Unadjusted model (reference).

Change percentage: Percentage change in model fit index relative to the crude model.

GVIF: Generalized Variance Inflation Factor. GVIF^(1/(2*Df)) < 2 indicates acceptable

multicollinearity.

Coeff: Regression coefficient from multivariable logistic regression with Intrinsic Capacity as the

dependent variable.
