# Supplementary material for: Association between aspirin use and decline in intrinsic capacity among community-dwelling elderly: a study based on the Lianyungang ICOPE pilot project
Source: Front Med (Lausanne). 2026 Jun 24;13:1769131. doi: 10.3389/fmed.2026.1769131 (PMC13341446; doi:10.3389/fmed.2026.1769131)
Supplement: Supplementary file 2 [file Image_1.pdf]

Supplementary Figure 1. Bias plot assessing the robustness of the association between aspirin use and decline in intrinsic capacity to unmeasured confounding.

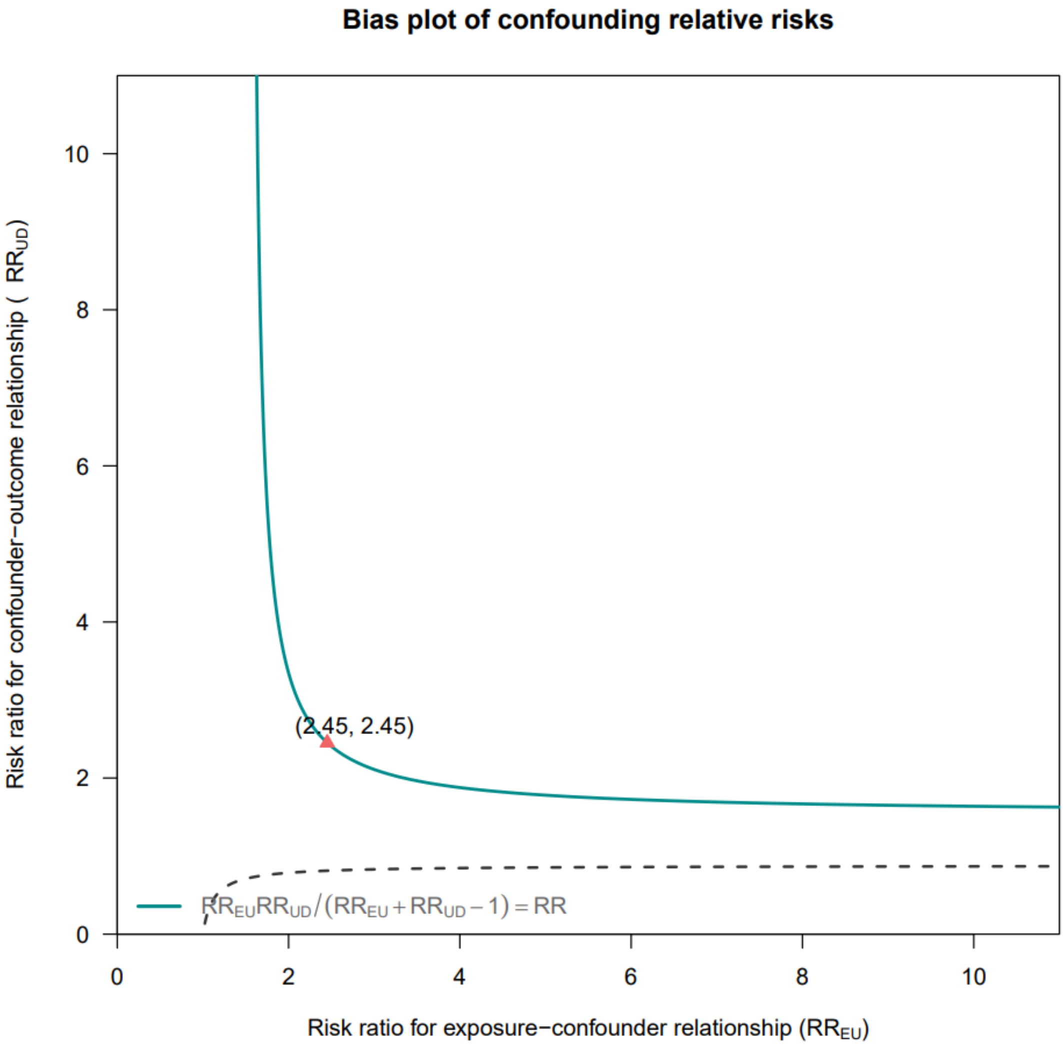

The solid curve delineates the boundary of relative risks (RRs) for an unmeasured confounder–outcome relationship (RR_ UD, y-axis) and exposure–confounder relationship (RR_ EU, x-axis) that would fully attenuate the observed association to the null (RR=1). The red triangle marks the E-value (2.45), representing the minimum strength of association an unmeasured confounder must exhibit with both exposure and outcome to completely eliminate the observed effect. This E-value indicates that an unmeasured confounder would need to have an RR of at least 2.45 with both aspirin use and intrinsic capacity decline to nullify our findings, supporting the robustness of our primary results.
